# An app-enhanced cognitive fitness training program for athletes: The rationale and validation protocol

**DOI:** 10.3389/fpsyg.2022.957551

**Published:** 2022-08-30

**Authors:** Eugene Aidman, Gerard J. Fogarty, John Crampton, Jeffrey Bond, Paul Taylor, Andrew Heathcote, Leonard Zaichkowsky

**Affiliations:** ^1^College of Sport and Exercise Psychologists, Australian Psychological Society, Melbourne, VIC, Australia; ^2^Division of Human and Decision Sciences, Defence Science and Technology Group, Edinburgh, SA, Australia; ^3^School of Biomedical Sciences & Pharmacy, University of Newcastle, Callaghan, NSW, Australia; ^4^School of Psychology, University of Southern Queensland, Toowoomba, QLD, Australia; ^5^College of Education & Human Development, Boston University, Boston, MA, United States

**Keywords:** cognition, fitness, performance, enhancement, intervention, wellbeing, technology

## Abstract

The core dimensions of cognitive fitness, such as attention and cognitive control, are emerging through a transdisciplinary expert consensus on what has been termed the Cognitive Fitness Framework (CF2). These dimensions represent key drivers of cognitive performance under pressure across many occupations, from first responders to sport, performing arts and the military. The constructs forming the building blocks of CF2 come from the RDoC framework, an initiative of the US National Institute of Mental Health (NIMH) aimed at identifying the cognitive processes underlying normal and abnormal behavior. Similar to physical conditioning, cognitive fitness can be improved with deliberate practice. This paper reports the development of a prototype cognitive fitness training program for competitive athletes and the protocol for its evaluation. The program is focused on primary cognitive capacities and subtending skills for adjusting training rhythms and enhancing readiness for competition. The project is driven by the Australian Psychological Society’s College of Sport & Exercise Psychology and includes the development of a Cognitive Gym program for a smartphone app-enhanced implementation. Its key building blocks are training protocols (drills) connected by a periodized training plan. A website with background supporting resources has also been developed as part of the project. National-level training squads will participate in a three-week pilot evaluation protocol, assessing the program’s efficacy and usability through gamified cognitive assessment of participants’ training gains and coaching staff evaluations, respectively. Both near and far transfer of training effects will be examined.

## Introduction

We live in a world that is increasingly dominated by technology. We feel its presence in our work, our leisure, and (for some) even in our sleep. Technology allows us to do some things that we could not otherwise do, almost invariably to do things more efficiently, and arguably in many cases to do things better. Perhaps the last frontier, the one place where we are yet to see a widespread influence of technology, is human cognition, the thoughts and feelings that accompany us as we go about our daily lives. Developments in technology, especially those associated with ubiquitous handheld devices such as mobile phones, open up new prospects of utilizing technology to improve the way we think, feel, and perform. The physical fitness arena offers a model of how technology can improve our physical wellbeing. Inexpensive apps deployed on a mobile phone and connected to wearable monitoring devices enable individuals to track progress toward performance goals. The setup is so unobtrusive that it is used by a growing number of fitness participants and professional athletes—both in training and during competition.

What made these things possible was the combination of advances in the knowledge of human physiology, the development of wearable sensors capable of detecting signatures of different body biomarkers, and the algorithms that translate these signals into meaningful and, more importantly, actionable insights. Similar advances are occurring in the cognitive domain, to the extent that it is now possible to monitor the brain’s electrical signals without wearing a cumbersome cap and to send this information back to the user *via* a mobile app that receives and translates the information into a meaningful form, e.g., Sherlin et al. (2013) and Mansour and Ouda (2019). This results in a level of biofeedback that will be surpassed only when we know more about the brainwave patterns themselves and their relationship with performance, both physical and mental. Such developments have substantial implications for athletic endeavor, where legitimate aids to performance are usually embraced.

### Apps for cognitive training

The type of mobile app described above represents a biofeedback device where the aim is to provide sophisticated forms of physiological feedback, including brainwave patterns. When we move into the cognitive domain, however, the potential uses for apps extend beyond giving feedback, rendering them potentially much more powerful tools for change. An app cannot be used to directly measure physical performance because the two domains are incompatible but that is not the case in the cognitive domain where an app can be used to both measure and train cognitive performance. It can do this by presenting cognitive tasks to participants and assessing their performance in these tasks as expression of psychological skills that are known in sport psychology to influence physical performance. The logic underlying this approach to app content is that by focusing on drills that build these psychological skills and prepare the mind and body for high performance, improvements in wellbeing and competition results will follow (e.g., Golby and Wood, 2016; Lane et al., 2016).

### Requirements for an effective app

Apps of this kind are more effective if they are based on a model of human functioning (e.g., Kletzel et al., 2016; Shah et al., 2017). The app described in this paper satisfies this requirement in that it is based on the Cognitive Fitness Framework (CF2: Aidman, 2017, 2020). The skills that are trained through the app should also be drawn from an established taxonomy of cognitive constructs covering the full range of human performance. In the present case, these constructs are contained in the CF2 model, which is itself based on the Research Domain Criteria (RDoC) framework (Morris and Cuthbert, 2012), an initiative of the US National Institute of Mental Health (NIMH) aimed at identifying the cognitive processes underlying both normal and abnormal functioning. Albertella et al. (2022) have extended the RDoC framework to cover cognitive constructs involved in high performance. An app of this kind should also be backed by additional resources, preferably online, that instruct the user in the operation of the app and offer additional guidance on the constructs and techniques that may be unfamiliar to some users.

Performance feedback is a key feature of “deliberate practice” (Ericsson and Harwell, 2019). This drives the requirement for measures of performance in the app to provide such feedback. These measures can take several forms. The first form of measurement will be the performance scores on the cognitive drills the user is performing. This requirement is easily satisfied when the drills take the form of objective tasks, such as an attention-switching task, where scores indicating errors or completion time produce feedback to the user. For an imagery or breathing task, on the other hand, rating scales can be more suitable. Measures of this kind capture performance on the app tasks but remain moot on how this performance translates to the real-life tasks that are the ultimate object of the training. Despite this limitation, there is still merit in measuring performance on the drills because they tap underlying constructs identified in RDoC and CF2 as fundamental to high performance. The app described here contains these inbuilt measures and records them in the form of a Leaderboard.

The final feature of the app to be described here is that it offers a prescribed training program of pre-set frequency and duration (in this study involving 30 min of daily practice with the app for 3 weeks) combined with a pre- and post-evaluation after a 3-week trial. The purpose of the evaluation is to assess both near and far transfer, the former referring to “increased performance on untrained tasks involving similar cognitive functions” and the latter referring to “increased performance on loosely related untrained tasks, or even activities of daily living” (Smid et al., 2020, p. 351). Our evaluation protocol assesses a form of near transfer by asking participants in the trial to complete a battery of cognitive tests developed by a research team not associated with the app project. These computer-based tests assess the same cognitive constructs that are targeted by the drills in the app (e.g., attention-switching). The expectation is that if the drills help the participants to develop their cognitive skills in particular areas, not only will performance on the drills themselves improve over the training period but also performance on these independent measures of the same construct. Demonstration that this kind of transfer occurs would constitute a strong argument that the learning goes beyond the boundaries of the app and is generalizable to related tasks. To measure far-transfer, the evaluation component of this project contains other measures in the form of a pre- and post-intervention ratings of relevant cognitive skills from users and coaches. The expectation here is that use of the app and its supporting materials will lead to better overall cognitive fitness and wellbeing.

## Identifying the skills to be trained

This introduction has provided a broad overview of the characteristics we have built into this first version of the app. In the sections that follow, we provide detailed descriptions of each of the elements associated with the app, beginning with the identification of the cognitive constructs to be trained.

The list of psychological skills sports psychologists consider important for high performance and wellbeing was established in the early days of the discipline and has not changed a great deal since. Under the heading of “Psychological Skills,” the index to Williams’ (1986) book on personal growth and peak performance lists anxiety, concentration, energizing, goal setting, imagery practice, relaxation, self-hypnosis, and thought control (p. 391). This original list has evolved through finer distinctions and concepts like “thought control” can cover sub-skills such as self-talk and thought stoppage Zaichkowsky and Peterson (2018). Terms like “relaxation” can cover sub-skills such as breathing, meditation, and progressive muscular relaxation. Indeed, these terms can all be found in the index of that same 1986 publication, each with associated training drills. Hammermeister et al. (2012) arrived at a broader set of psychological skills in their search of the sport literature. Their list included confidence, sport intelligence, the ability to focus, competitiveness, a strong work ethic, goal-setting abilities, coachability, high levels of hope and optimism, and adaptive perfectionism.

Selecting relevant skills and associated training drills for inclusion in an app is not a straightforward task. A further guiding principle we adopted was that, wherever possible, we searched for the “cognitive primaries”, the fundamental capacities that underpin multiple psychological skills.

### The research domain criteria framework

The factor analytic literature was a possible source of this information but there are no factor analytic studies that cover the full range of psychological skills involved in performance. That task would require the type of massive survey undertaken by Carroll (1993) but extended even further to include personality, confidence, and other constructs that come under a more loosely defined “cognitive” label. Instead, for this part of the project, we turned to the RDoC (Morris and Cuthbert, 2012). The RDoC initiative, sponsored by the US National Institute of Mental Health (NIMH), grew out of a desire to provide a research framework “that encourages investigators to reorient their research perspective by taking a dimensional approach to the study of the genetic, neural, and behavioral features of mental disorders” (Morris and Cuthbert, 2012, p. 29). By adopting a dimensional approach to mental disorders, researchers no longer viewed those suffering mental disorders as belonging to distinct categories, an unfortunate outcome when using classification systems such as the Diagnostic and Statistical Manual of Mental Disorders (DSM) and the International Classification of Diseases (ICD)—but as normal people whose symptoms are due to abnormal levels of common, underlying psychological traits and neurobiological conditions. Of interest to us, as we searched for elements to include in our app, was the broad sweep of this new framework. “RDoC’s integrative approach includes cognition along with social processes, arousal/regulatory systems, and negative and positive valence systems as the major domains, because these neurobehavioral systems have all evolved to serve the motivational and adaptive needs of the organism.” (Morris and Cuthbert, 2012, p. 29).

The underlying philosophy of RDoC is a good match to our project because psychological skills lie on a continuum and although we use the term “elite” to describe athletes performing at the highest level, most sport psychologists see elite athletes as normal people with highly developed physical talents and psychological skills. The CF2 model (Aidman, 2020) is also based on this philosophy. It considers both mental health and high-performance as “natural consequences of the varying levels of psychological functioning (including cognitive, affective and motivational) ranging from deficit to norm, and further to high or gifted performance.” (Aidman, 2020, p. 3).

More importantly, RDoC offered substantial benefits for this project from a practical point of view. RDoC identified broad higher-level domains of functioning that comprise multiple sub-dimensional constructs, reflecting state-of-the-art knowledge about major systems of cognition, motivation, and social behavior. The latest version of the framework can be found at https://www.nimh.nih.gov/research/research-funded-by-nimh/rdoc/constructs. It takes the form of a matrix with the rows comprising six major domains, each with a number of subconstructs, and the units of analysis, of which there are eight, comprising the columns. Table 1 shows the domains, the constructs, and the subconstructs that have been identified to date.

**Table 1:** A simplified representation of the RDoC matrix.

Domains	Constructs	Subconstructs
Negative valence	Acute threat Potential threat Sustained threat Loss Frustrative non-reward	FearAnxietyGriefFrustration
Positive valence	Reward responsiveness	Reward anticipation Initial response to reward Reward satiation
	Reward learning	Probabilistic and reinforcement learning Reward prediction error Habit
	Reward Valuation	Reward probability Delay Effort
Cognitive systems	Attention	
	Perception	Visual perception Auditory perception Olfactory/somatosensory/multimodal perception
	Declarative memory	
	Cognitive control	Goal selection, updating, representation, and maintenance Response selection, inhibition/suppression Performance monitoring
	Working memory	Active maintenance Flexible updating Limited capacity Interference control
Systems for social processes	Affiliation and attachment	
	Social communication	Reception of facial communication Production of facial communication Reception of non-facial communication Production of non-facial communication
	Perception and understanding of self	Agency Self-knowledge
	Perception and understanding of others	Animacy perception Action perception Understanding mental states
Arousal/regulatory systems	Arousal Circadian rhythms Sleep and wakefulness	

Adapted from Albertella et al. (2022).

As can be seen in Table 1, this simplified view of the RDoC matrix comprises six domains, 25 constructs, and 32 subconstructs. As such, it offers a compact representation of RDoC, with its original rows presented as three columns. In addition to the constructs shown in Table 2, the RDoC matrix distinguishes seven levels of measurement granularity for each subconstruct, from Genes, Molecules, Cells and Circuits, to Physiology, Behavior, and Self-Report, with a collection of known methods of measuring the RDoC constructs at different levels of measurement listed under the Paradigms column. The cells of the matrix were of particular interest to the present project because they give some indication of measures that can be included in an app to measure training gains in the chosen cognitive capacity or skill.

**Table 2:** Training targets in the Cog Gym 1.0 App: Modified from Aidman’s (2020) phases of the cognitive fitness cycle.

Phase	Domain of cognitive functioning	Target constructs	Examples of training/development objectives
Foundational training	Cognitive fitness	Self-awareness	Stress symptoms detection
(Cognitive gym)	Trainable cognitive primaries	Attention	Focus endurance
			Focus control: breadth & direction^*^
		Task switching	Dual-tasking
Advanced cognitive training	Cognitive skills	Controlled response	Effortless concentration
		Energy management	Arousal regulation
			Resonant frequency breathing
		Situation awareness	Sense-making (interpretation)
			Anticipatory skills (prediction)
		Decision making	Pattern recognition
			Confidence calibration
		Adaptability	Cognitive flexibility
Mission-ready training	Tolerance and resistance	Tolerances	Generalized discomfort tolerance
			Mental effort tolerance
			Frustration tolerance
		Resistances	Distractor resistance
			Susceptibility to deception
		Task resilience	Error detection
			Performance recovery
Operational augmentation	Operational task performance	Cognitive state	Alertness monitoring
		Cognitive Workload	Fatigue countermeasures
Recovery	Cognitive recovery	Reflective practices	Mindfulness and meditation

* Newly added construct.

Constructs selected from Aidman’s (2020) Table 1.

### RDoC extension to high performance

The RDoC constructs replace symptom clusters with measures of root causes of the underlying dysfunction (e.g., Yücel et al., 2019). Extending their range of measurement from dysfunction and clinical populations to the well-adjusted and high-functioning individuals is an important next step, given that nonclinical populations have so far been under-represented in the RDoC-driven research. Making this transition to the upper range of functional capacity may also help reframe considerations of “mental health” into ones of “mental fitness” by shifting from the stigmatized “shall I seek help/treatment?” to the more positive and proactive “can I train for it?”

A transdisciplinary Delphi study (Albertella et al., 2022) examined the applicability of the model to the high-performance domain using the same methodology that was employed in the development of the original RDoC. We used a three-stage process to achieve this aim. The first stage involved establishing whether RDoC constructs covered the whole of the performance continuum. That is, whether its constructs were adequate to describe high performance or whether new constructs were needed. The second stage involved the identification of measures for constructs involved in high performance. The third stage involved the identification of tasks to train. All three stages were important for the construction of the app and associated supporting materials. The methodology used in the three stages of this project is described in more detail in the paragraphs that follow.

A strong feature of RDoC is the use of expert panels to revise and expand the set of constructs and subconstructs. Accordingly, an expert consensus was sought on the relative importance of primary RDoC constructs and their subconstructs to various high-performance applications. This consensus built on the RDoC foundational evidence in defining major domains for the study of cognitive fitness and developing guidelines for assessing them using an optimal mix of biomarker, physiological, behavioral, and self-report measures. It was expected that the project would inform the development of measurement and assessment protocols for these dimensional constructs and lead to tailored training programs aimed at maximizing performance and longevity for the workforce in demanding occupations. The expert consensus-building study included two main components: an expert advisory group phase, which developed guidance on the content and direction of the Delphi study, and the Delphi study itself.

The Delphi study is described elsewhere (Albertella et al., 2022) but its main findings are worth recapping here because they were used as input when designing the content of the app. The key question presented to the experts throughout the Delphi method was as follows: “How important do you think a construct (e.g., attention) is to *optimal performance* in *dynamic* and *high-pressure* environments?” This question and corresponding *key term* definitions were decided through discussion with the advisory group that included experts in military and sport psychology, as well as in high-stakes civilian applications such as paramedic and emergency services. Specifically, Albertella et al. (2022) defined *optimal performance* as performance that successfully achieves an individual’s goals, consistently (i.e., not by chance). Constructs could reflect preparation and recovery aspects of performance in so far as these could influence the likelihood of consistent, optimal performance, as well as the “in-the-moment” performance execution. Finally, such performance could apply to any level of expertise, that is, from novice to elite. *Dynamic environments* were defined as contexts that had the capacity to change and do so inconsistently and unpredictably. Building on the *performance under pressure in sport* literature (Jackson et al., 2006; Arrondel et al., 2019; Ötting et al., 2020), the definition of *high-pressure environments* was extended to less benign occupational contexts where the stakes are much higher (life and death), the rules are less defined and scenarios involve multiple concurrent operations (military and fire fighters). As a result, two categories of *high-pressure environments* were defined. In the first category were environments characterized by high risk or capacity for significant loss or gain, including life-or-death consequences, “high visibility,” or “high expectation.” In the second category were environments characterized by “high demand,” such as complex scenarios requiring multiple ongoing concurrent operations.

In brief, the study produced a transdisciplinary consensus on ten cognitive factors relevant to high performance, including: (1) Attention; (2) Cognitive Control: Performance Monitoring; (3) Arousal; (4) Cognitive Control: Goal Selection, Updating, Representation and Maintenance; (5) Cognitive Control: Response Selection and Inhibition/Suppression; (6) Working memory; Flexible Updating; (7) Working memory: Active Maintenance; (8) Perception and Understanding of Self—Self-knowledge; (9) Working memory—Interference Control; and (10) Shifting. Seven of the ten constructs that reached consensus across all four Delphi panels came from RDoC’s Cognitive Systems domain. The remaining three constructs came from Social Processes, Arousal, and Regulatory Systems domains, or were added to the RDoC construct set by the experts.

This expert consensus was seen as instrumental to standardizing cognitive assessment and informing mechanism-targeted interventions in the broader field of human performance optimization. In particular, it gave the research team the opportunity to evaluate the suitability of the CF2 model, which is based on the RDoC framework, as a platform for the mobile app.

### The cognitive fitness framework model

**Figure 1:**
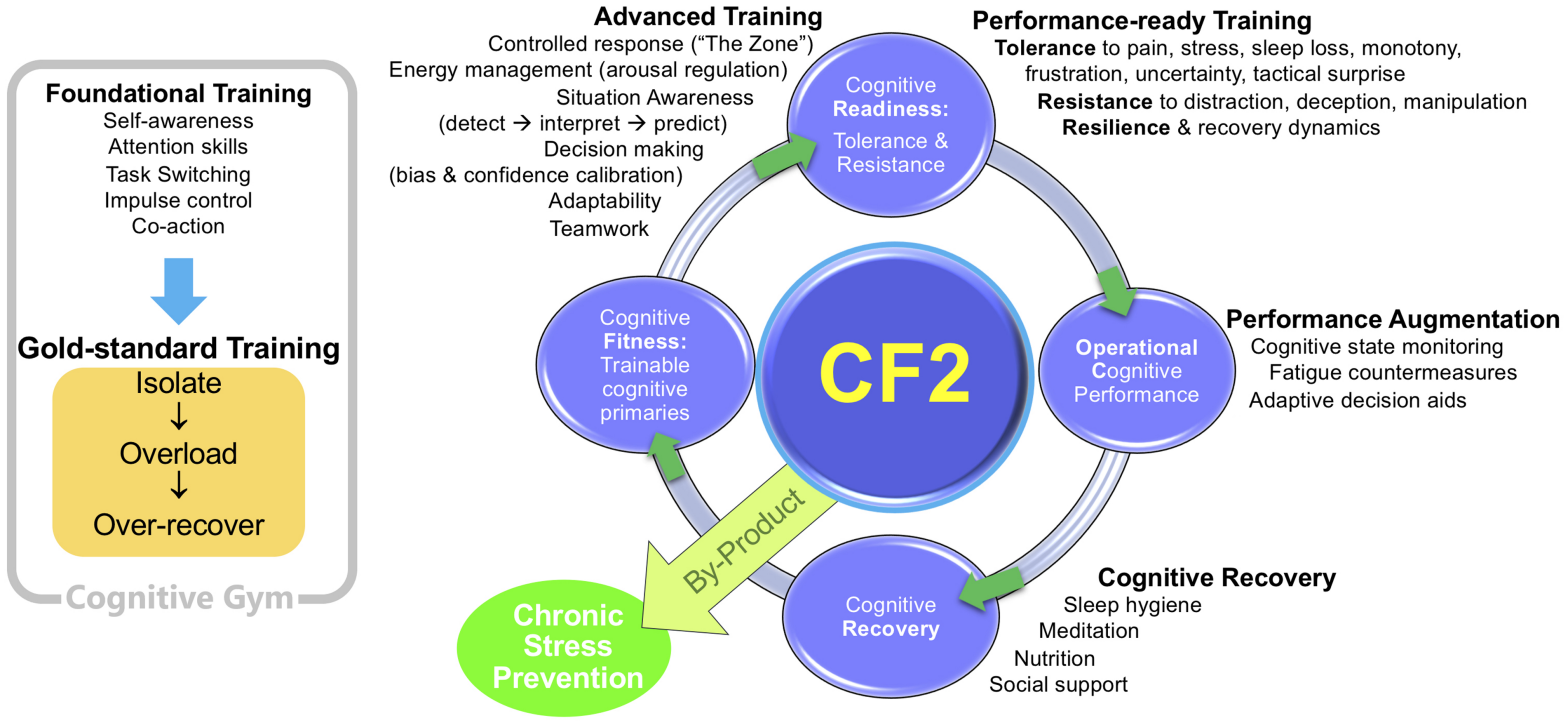
The Cognitive Fitness Framework (CF2; adapted from Aidman, 2020).

The CF2 model represents a holistic, integrated view of the cognitive factors underlying high performance. Figure 1 shows an adaptation of Aidman’s (2020) original version.

A major feature of the model is the transition through stages, showing how different levels of cognitive fitness can be achieved and maintained. The Foundational Training leads to a state of Cognitive Fitness. From there, the model progresses through Advanced Training, Performance-ready Training, Performance Augmentation, thence to a Cognitive Recovery phase. Not only is there is a gradual build-up of skills in CF2, there is also a development of particular skills for particular situations. In the same way that the body has different muscle groups which require different exercises, different mental exercises improve different aspects of the mind. Some mental exercises improve focus, some help to reduce stress and anxiety, some can sharpen self-discipline and increase motivation, and some aid recovery.

The link between phases, domains of cognitive functioning, RDoC target constructs, and training objectives is spelled out more completely in Table 2. This table has been adapted from Aidman (2020) by selecting the constructs that we deemed fitting with the prototype training intervention in the current study. Following the same logic, we have added one extra construct, “Focus control: breadth & direction.”

The CF2 model is already being used in workplace applications aimed at improving mental health and wellbeing (e.g., Taylor et al., 2021). Taylor (2021) used a combination of CF2-informed interventions to achieve improvements in mental wellbeing and resilience and a reduction in burnout for 800 workers in a range of businesses in the Australian corporate sector. The College of Sport and Exercise Psychologists (CoSEP) of the Australian Psychological Society (APS) is one of several stakeholder groups who have adopted the CF2 as a new paradigm in the management of the Mental Health—Performer Wellbeing—Performance Support operating environment. CoSEP identified a pressing requirement to support athletes and their support teams severely affected by the COVID-19 disruption to the sports industry. A prototype cognitive fitness program for competitive athletes has been developed, focused on fundamental mental capacities and subtending skills for re-setting and adjusting training rhythms and improving mental readiness for competition (Bond et al., 2020). In its original form, the prototype program generated promising user acceptance and training outcomes, according to coaches’ qualitative feedback. These early findings led to the consideration of a mobile app that could put the means for improvement in the hands of individual users.

## Prototype intervention

Table 2 summarizes the range of cognitive constructs and training objectives that could be addressed by a mobile app. When one considers that it takes sustained practice to acquire mastery of any of the elements in the last column of Table 2, it becomes evident that the range of training targets is far too large for any app. Our review of sports-related apps currently on the market suggests that most of them are modest in their offerings. They provide feedback on a narrow range of biomarkers and/or they concentrate on a limited range of skills (e.g., controlled breathing). Faced with this same dilemma for the initial version of our app, we decided to develop web-based resources that users could consult to gain a better understanding of psychological skills underpinning high performance and how they can be trained using the app. These web-based resources are more than an optional add-on, they form an essential component of the intervention, and will be described next.

### Building the app: Web materials

The CF2 App is supported by a website designed to provide additional support for the study participants. This support takes the form of:

- an educational video used as a pre-briefing to the app-driven training program, looking at developing a “High Performance Mind Set,” establishing a strong “Self-Belief,” and better understanding of “Attentional Control;”
- an educational video covering skills involved in the training program;
- additional reading expanding on the skills involved in the training program;
- additional reading providing contextual case studies of athletes being challenged in competition to use the skills involved in the training program.

There are ten pages on the website. The Home page provides a simple “concierge” function that provides participants with direct links to the pre-briefing videos referred to in their introductory correspondence.

The second page introduces the Cognitive Fitness Framework. The messages and definitions are provided in a language system that is compatible with the app and the pre-briefing videos. It provides participants with logical progressions of the concepts behind the training program.

The third page carries the title “How This Works,” and it provides an explanation of how the app and the website work together. It provides a “Road Map” of where the participants should go for various aspects of the study.

Page 4, headed “Elite Systems,” has two sub-pages: “Standard Practice Routines” and “Manage Your Mental Health.” This section provides information on the skills, attitudes, and capabilities that individuals with strong cognitive fitness evidence. It also explains the basis of the routines and origin of the systems behind the training program structure. The section also addresses the links between “Cognitive Fitness Training” and mental health management.

Page 5, headed “Briefing,” has four key sub-pages, and additional topic support sub-pages. The key sub-pages are:

- “Mind-Set and Self-Belief Principles Embraced by Elite Performers.”
- “Mind-Set and Self-Belief Background Material.”
- “Attention Control Principles Embraced by Elite Performers.”
- “Attention Control Background Material.”

The additional topic support sub-pages include “Imagery,” “Relaxation,” and other areas that sub-tend some of the drill structures.

The key sub-pages referring to principles embraced by elite performers contain the pre-briefing video material that participants are required to interact with before commencing their training program *via* the app.

Page 6 is headed “Definitions,” and contains a glossary of terms used both in the Website and the app. It is designed to facilitate participants progressing through their training program with an unambiguous explanation of key concepts.

Page 7 is headed “References,” and contains a systematic collection of links to additional materials and sites relating to the concepts contained in the website and the training program contained in the App.

Page 8 is headed “Stories” and contains case study examples of athletes requiring or using the skills developed in the training program. These case studies inform participants’ understanding of the relevance of the drills that comprise the training program.

The last page is headed “More.” It contains links to other resources, such as papers, infographics or talks on topics of interest to those seeking to improve performance or overall wellbeing. Examples include special breathing techniques, heart rate variability, performance debriefing, and how to better cope with the lifestyle demands of performing in high expectation environments.

Although the website adds some further resources for those looking for ways to improve wellbeing and performance, at its core are the pages supporting the app, which we will now describe.

### Cognitive gym: Target behavioral outcomes

**Figure 2:**
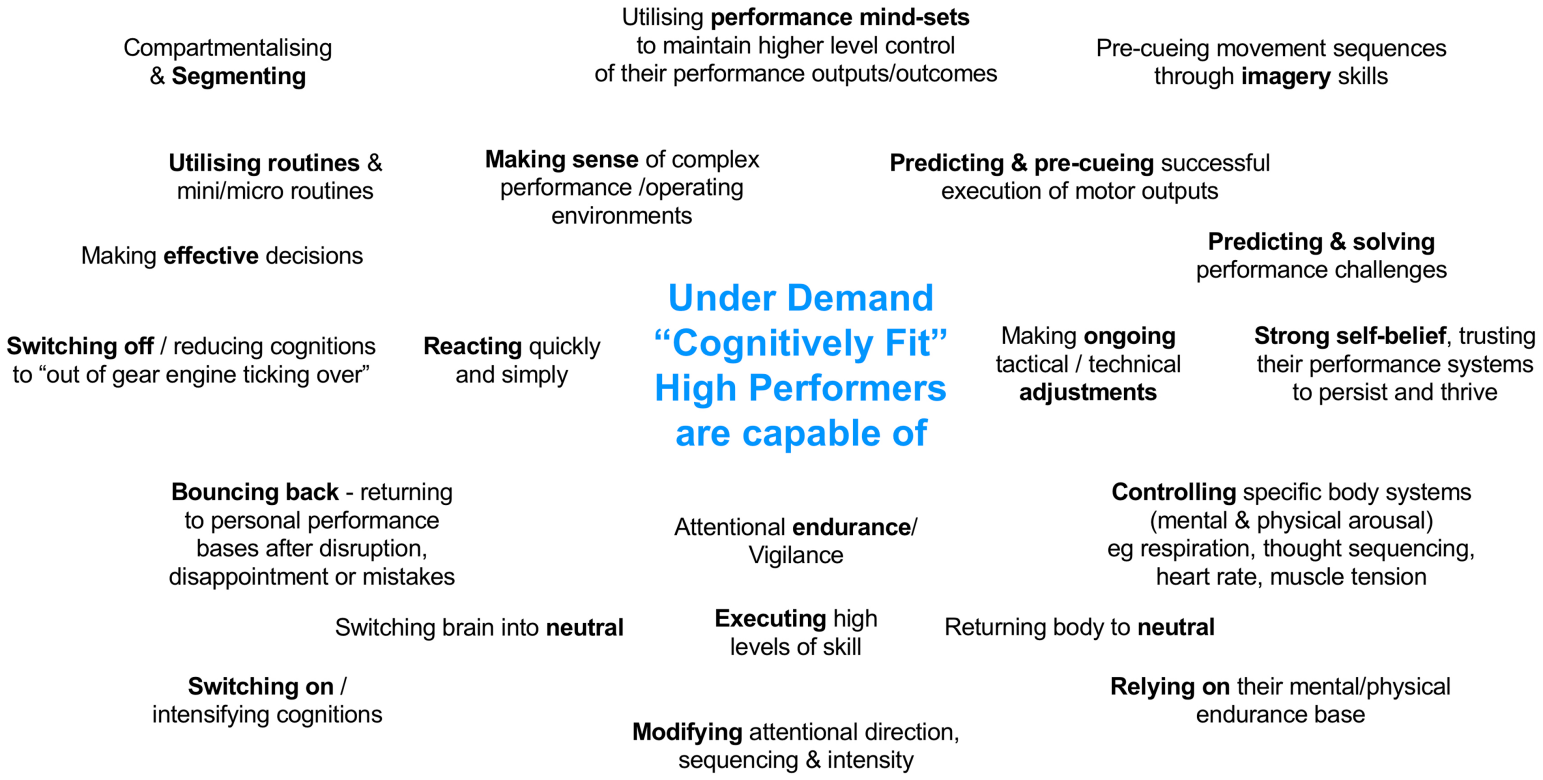
Functional capabilities of high performers (adapted from Crampton et al., 2021).

A number of decisions had to be made before work could start on the app. Moving some of the supporting material to the Web was the first of those decisions. Deciding not to offer biofeedback in the first version of the app was the second decision. The most difficult decisions, however, concerned the choice of cognitive abilities and associated drills to include. To help with that task, we drafted a set of expectations of high performers that was consistent with the CF2 model. Those expectations are shown in Figure 2.

Figure 2 provides verbal descriptions of behaviors that we are seeking at various stages of the performance cycle. To select from the many construct/drill combinations that are available, we then devised a filtering system that we could apply to any drill (Crampton et al., 2021). The first step of this filtering system involved what we call a “first-glance” evaluation of the drill where we examined its broad qualities, such as whether it was suitable for inclusion in an app. The second step applied a more stringent cause-and effect evaluation to check whether the drill is supported by research. The third step considered whether the drill could produce reliable and valid measures of the construct it is developing. The fourth step considered the availability and quality of instructions for the drill. Do these exist? Are they adequate? If not, can they be developed in a form that will suit the app? If the drill passed through each of the filters, it became a candidate for inclusion in the app. The same filtering system can be applied to constructs as well as drills, even those selected from the CF2-based Table 2.

After selecting the drills for inclusion in this prototype, the next decision related to ways of presenting them. Options included stand-alone drills or combinations of drills in the form of loops or sequences. A concentration drill, for example, may be interspersed with breathing exercises, in much the same way that an athlete would be expected to use combinations of drills in training and competition settings.

### Cognitive gym training program: Practice drills

The initial version of the app was designed to satisfy a “proof of concept” requirement for the project. For this purpose, the selected drills were combined into a single intervention to form a Cognitive Gym. The intervention employs a blended delivery methodology, combining face-to-face pre-brief with a three-week program *via* a web application that is designed to help participants exercise their primary cognitive capacities and performance-focused cognitive skills. The web application contains educational videos, guided cognitive workouts, breathing sessions, user engagement tools, completion trackers, leaderboards, and a social feed. The Cognitive Gym also uses gamification techniques to consolidate behavior change.

The current prototype (see Supplementary material S2, panels A,B) contains a recommended sequence of 2 h of instructor-led learning followed by one-and-a-half to 3 weeks of 30-min daily practice. This core sequence includes:

1. Program “brief in” introducing the intervention as “cognitive fitness training” similar to conditioning and skill training work. Included in the briefing is an education section on developing a “high performance mindset” and on developing understanding and control of attentional flexibility. Participants will be encouraged to apply their knowledge of those areas to their cognitive fitness work. This two-hour instructor-led instruction was accomplished by preparing a set of eight introductory videos promoting the use of the app to achieve a high-performance mindset. The additional Web resources described earlier are also available to the trainee.
2. Daily 30 min practice involves ten sequential drills, guided by a mobile device-based app with built in engagement functionality to encourage participants’ continued involvement. The ten drills are described below.

- External attentional flexibility: Participants are guided to utilize the camera on their mobile device to rapidly shift their focus from narrow to medium to broad external awareness of their surroundings for 45–60 s.
- CubeRunner 2: simulated aircraft flight through congested, distracting airspace. Participants are instructed to maintain a flexible external focus of attention for extended periods on a gamified cognitive control task, notified by a three-minute countdown timer. This task is illustrated in Supplementary material S2 (panels D and C).
- Do not be in a hurry to breathe: Participants are guided through a breath-based relaxation induction followed by a challenge to reduce breathing rate to the lowest comfortable cadence and sustain the pattern for a string of ten breathing cycles focused on slow and smooth transitions from exhale to inhale. The drill finishes when focus is lost, after which the participant completes the remaining attempts as practice, and is then guided through a breath-based “wake up” exercise returning awareness and breathing to baseline.
- Color timer: Participants maintain an extended narrow external focus of attention and respond by tapping a colored dot matching a color dot stimulus in a three-choice reaction task. Stimulus presentation is repeated in an ongoing random sequence with a specified time limit for each response. Missing the allowed time finishes the trial, resulting in a string score (number correct within time in a row). The aim is to produce a new personal best string each trial. Multiple trials are permitted within the allocated drill time. A countdown timer is used to advise of the end of the 3 min of training.
- Getting centered: Participants are guided through a sequence of thoughts and feelings that build a sense of control of their center of gravity—incorporating postural and specific breathing instructions.
- Higher lower: Participants complete a simple numeric operation on screen and respond by swiping the screen up or down to indicate whether the result of their calculation is higher or lower than the number previously presented on screen. The response is timed as well as scored for accuracy.
- Empty headed: Participants are guided through an imagery-based relaxation induction followed by a sequence of trials where they attempt to “let go” of distracting thoughts across varying periods of time. The time periods are directed by an audio recording. The three-minute drill concludes with an imagery-based “wake up” exercise.
- Red square: A three-minute collision-avoidance task where participants are required to maintain a flexible external focus in order to move their avatar (a red square) away from three randomly moving blue squares. The aim is to improve personal best scores (expressed in seconds) across each trial. Multiple trials are permitted within the allocated training time. The end of the training period will be notified by a countdown timer.
- Imagination sequence: Participants will be guided through a three-minute sequence of imagery training drills. The sequence commences with a loop of generic drills, and then progresses to a sequence of imagery drills related to aspects of their training or competition performances.
- Attentional spiral: Participants will be guided through a three-minute sequence of attention shifts that move from generic to personally relevant cues, and then they are assisted to transition to their ongoing daily challenges.

The drills are the building blocks of the Cognitive Gym. They will be administered either alone or in combination (e.g., imagery drills preceded by breathing drills). Combinations comprise as many as five different drills arranged as loops, sequences, spirals, or other sequences. Each drill involves systematic and disciplined execution of underpinning cognitive skills, such as concentration, endurance, and attentional flexibility. The core instruction is delivered *via* the smartphone mobile app (see Supplementary material S2, panels A and B) and backed by the companion website described above, which offers extensive background information and additional practice options.

## Evaluating the cognitive gym prototype: Methods

It is important to evaluate the prototype app in the context of a well-defined Cognitive Gym training package catering for a well-defined user group. The current proof-of-concept study will evaluate the app-supported Cognitive Gym training package in a sample of competitive athletes. The evaluation will assess training gains and participants’ engagement with the app. More details are given below.

### Participants

A total of 60 participants will be drawn from national training squads using the project team’s coaching contact lists (see sample size calculations in “Planned analyses”). All members of the selected training squads aged between 18 and 30 years will be invited to participate but they will be free to decline or withdraw at any point without any adverse consequences. Younger athletes will be excluded from the current study, with a separate study planned to address this age bracket. Participant IDs will be issued to all consenting athletes. The dataset will include demographic data including age, gender, sport and functional role, but it will contain no personally identifiable information.

### Study design

A cross-over design was chosen for this study as it improves statistical power by making each participant a baseline for themselves. It is also driven by the need to achieve sufficient buy-in from the participants and their coaches—the groups known for their dislike for passive control designs where participants in control conditions can miss out on the potentially beneficial intervention.

In our cross-over design participants will be randomly assigned to either a “training-first” or “waiting first” group. All participants will complete baseline assessment using methods (described in “Outcome measures”) at the start of Week1 (Time 1), then repeat it at the start of Week 4 (Time 2) and at the completion of Week 6 (Time 3). A single blind strategy will be utilized, with researchers administering pre- and post-assessments having no access to the information on which group the participants belong to (training-first or waiting-first).

Following Time 1 assessment, the “training-first” group will commence the three-week training program described in Section 3.3 above, spending 30 min a day practicing the drills delivered through the Cognitive Gym app. The “waiting first” group will have no engagement with the Cognitive Gym app for the first 3 weeks but will have access to the companion website for the designated 30 min daily. They will commence their three-week training with the app immediately after Time 2 assessment. Access to the app will cease for the “training-first” group after Time 2. As a result, each participant will go through 3 weeks of Cognitive Gym app-supported training and 3 weeks waiting, with half of them randomly assigned to training first and then waiting, and the other half—to a reverse order. Coach ratings of athlete progress will be examined in the evaluation, so their consent to participate will also be sought.

### Outcome measures

The efficacy of the Cognitive Gym protocol will be assessed with (1) pre- and post-intervention cognitive testing administered *via* CogMission online platform (Wells et al., 2021; Kucina et al., 2022), (2) athletes’ self-ratings of their typical cognitive performance over the preceding 3-week period, and (3) coach ratings of athletes’ performance in their sport-specific training to evaluate the all-important far transfer.

#### CogMission platform

The CogMission online testing platform (Wells et al., 2021; Kucina et al., 2022) delivers a testing protocol based on a gamified dynamic scenario containing trials made up of the following three tasks, all requiring a binary response by pressing either the Left (z) or Right (/) key on the keyboard:

- Working memory basic task. Participants are asked to remember which of three doors was highlighted two trials ago and what symbol (0/×/+) is present on the door, then use that information to decide if the door highlighted on the current trial matches.
- Stop-signal task. This is a classical task used to measure response inhibition (Matzke et al., 2018). An arrow appears on the highlighted door and the participant notes which direction it points. If a red outline appears around the door after the arrow appears (25% of trials) the participant must withhold their response. The stop-signal delay (from the onset of the arrow to the red frame over the door) is adjusted over trials to ensure that stopping is successful on approximately 50% of trials.
- Selective attention task. The task measures the ability to control interference from irrelevant spatial and verbal information using Stroop (MacLeod, 1991) and Simon tasks (Hommel, 2011), and information from irrelevant spatial locations using a Flanker task (Eriksen, 1995). The door opens, the room behind is shown, a cue at the top of the screen indicates the side where opposition is hiding, and the participant must press the corresponding button. Cues can be the direction of a central arrow (surrounded by flanking arrows: left = <<<<< or > > <>>, right = >>>> > or > > <>>) or the color (e.g., left = blue, right = orange) of a rectangle or word (“BLUE” or “ORANGE”). Two such decisions are made in sequence on each trial, sometimes with the same type of cue and sometimes with different cues. This allows measurement of cognitive flexibility in terms of updating goals using a task switching paradigm (Monsell, 2003). That is, on each trial participants perform two conflict task trials in a row. When the two tasks are the same, the second trial does not require updating the goals established by the first (a “repeat” trial). If the two tasks differ the second trial requires updating of goals (a “switch” trial). Slower response times on switch trials relative to repeat trials indicate reduced flexibility.

An important feature of this protocol is that it combines the Stroop task with Simon task. Stimuli (the words BLUE or ORANGE presented in either a blue or orange font) appear on either the left or right of the screen. Participants respond to the color of the word (e.g., pressing an “orange” key on the left or a “blue” key on the right). Stroop interference is produced when, for example the word BLUE is presented in orange. Simon interference is produced when the word appears on the opposite side to the key indicating the color of the word (e.g., pressing the right key for a blue word that is presented on the left). This combination has been shown effective in reducing the testing time and improving the reliability of the resulting individual differences measures (Wells et al., 2021; Kucina et al., 2022).

Participants initially perform a short (20-min) self-paced tutorial to familiarize themselves with each of the tasks. Participants then perform 16 sets of 12-trial “games” taking self-paced rest breaks between each game. To maintain motivation and engagement, points are awarded for correct choices with bonuses for fast responding. The resulting measures include working memory, response inhibition, and cognitive flexibility.

#### Athlete self-ratings

Cognitive Fitness self-assessment, a set of 20 self-rating scales assessing the individual’s perception of their typical cognitive performance over the preceding 3-week period. Details are in Supplementary material S1.

#### Coach ratings

Coach assessment of “far transfer”: to ascertain the extent of training gains transfer to the sporting contexts, athletes will be rated by their coaches. Coach ratings assess the athlete’s attributes such as composure in competition (e.g., 1 = cool, 2 = calm, 3 = worried, 4 = flustered) and adaptability (e.g., 1 = rigid; 2 = gets stuck; 3 = flexible; and 4 = fully adaptive). More details are in Supplementary material S1.

### Planned analyses

It is expected that, with practice, participants will improve their performance scores on the respective drills. These changes will be monitored and evaluated throughout the intervention. A single blind strategy will be utilized, with researchers administering pre- and post-assessments having no access to the information on which crossover group the participants belong to (training-first or waiting-first).

Improved scores on the drills, however, is no guarantee that participants have learned skills that transfer to similar cognitive tasks (i.e., near transfer), such as the working memory, response inhibition, and cognitive flexibility tasks included in the CogMission battery. Near transfer effects will be evaluated by estimating training gains defined as differences between each CogMission measure at baseline and at the end of the Cognitive Gym training.

Repeated measures ANOVA and mixed linear modelling will be used to estimate effect sizes. Effect size of *d =* 0.4 on training-induced change is considered meaningful and practically important, based on a meta-analysis of randomized controlled trials examining the effects of similar programs on stress (Dhir et al., 2021). This effect size corresponds to *f =* 0.25 for ANOVA power calculations. To reliably detect effects of this size, with two groups and three repeat measurements in our design, G*Power calculations return a minimal sample size of 28 participants (Faul et al., 2009). Allowing for sample attrition and the uncertainty associated with extrapolating power calculations for mixed linear modelling, this study will aim to recruit 60 participants.

Far transfer effects will be evaluated through the coach and athlete ratings. Despite some well-known limitations of self-reports, they have advantages such as easy interpretability, information richness, and practicality (Paulhus and Vazire, 2009). Similar ANOVA and mixed linear modelling will be applied to assess training gains on these measures. Evidence of both near and far transfer effects will address the frequently-voiced objections that gamification techniques lead to improved performance in the games themselves but not in the broader skills they are meant to develop.

## Concluding comments

There is no shortage of apps in the marketplace claiming to support mental health and wellbeing. Performance focused apps are rapidly catching up. One of the key differentiators in both app types is the quality of evidence backing their protocols and claimed benefits. Our decision to develop a new app was guided by the following considerations:

- the app should be based on sound research evidence that includes a model of cognitive functioning (we chose the CF2 as our model);
- the app should target constructs that are known to influence actions at different stages of the performance cycle;
- the app should use practice drills that have passed through a series of rigorous filters;
- the app should be backed by a comprehensive psycho-educational package that provides instructional and motivational resources to the user; and
- the app should be evaluated against external criteria that would allow assessment of both near and far transfer of training.

These are the criteria that guided the development of the app. It is not yet possible to compare the performance of the app against other cognitive training software platforms. Reviews of these platforms are available (e.g., Kletzel et al., 2016; Shah et al., 2017), addressing some of the points covered above. A major difference between these platforms and the app we have described is the app’s focus on the upper end of the performance continuum and its reliance on supporting web-based psychoeducational tools that address the all-important domains of confidence, self-belief, and a mastery versus outcome focus.

Finally, it needs to be emphasized that this is an evolving program. The evolution of the Cognitive Gym training package, the app and its supporting materials will be informed by the evidence of the current prototype’s efficacy and user acceptance. The study protocol presented here is the first step in that direction.

## Data availability statement

The original contributions presented in the study are included in the article/Supplementary material, further inquiries can be directed to the corresponding author.

## Ethics statement

The drills included in the daily 30-min practice sessions require mental effort but pose no other risks exceeding inconvenience to participants. The evaluation protocol described here has been reviewed and approved by the Low Risk Ethics Panel constituted by Australia’s Defence Science and Technology Group (DSTG) under the National Health and Medical Research Council’s guidelines (Protocol Number LD 05-22).

## Author contributions

EA, JC, and LZ developed the Cognitive Gym concept. PT designed the core functions of the app. JC, AH, and JB developed the evaluation protocol. All authors conceptualized the paper and reviewed the drafts developed by GF and EA. All authors contributed to the article and approved the submitted version.

## Funding

This study forms part of a project funded by the Australian Army Headquarters (PO4501102378).

## Conflict of interest

The authors declare that the research was conducted in the absence of any commercial or financial relationships that could be construed as a potential conflict of interest.

## Publisher’s note

All claims expressed in this article are solely those of the authors and do not necessarily represent those of their affiliated organizations, or those of the publisher, the editors and the reviewers. Any product that may be evaluated in this article, or claim that may be made by its manufacturer, is not guaranteed or endorsed by the publisher.
