# Supplementary material for: An app-enhanced cognitive fitness training program for athletes: The rationale and validation protocol
Source: Front Psychol. 2022 Aug 30;13:957551. doi: 10.3389/fpsyg.2022.957551 (PMC9469727; doi:10.3389/fpsyg.2022.957551)
Supplement: Supplementary file 1 [file Data_Sheet_1.PDF]

# Supplement S1

# User Rating Scales for the Pre- & Post- Training Assessment

**Pre-Training Instructions**

**Rating Scales – introduction**
Cognitive Fitness is about how well prepared and "fit for purpose" your thinking systems are for your current challenges.

This set of rating scales are a way for you to identify / score how you have been feeling and how effectively you have been functioning over your last 3-4 weeks (when training, competing, or at work / when studying).

Use the 7-point rating scales for each of the following aspects of cognitive fitness - there are options for in-between ratings. Choose the number that best represents how you have felt.

| 1 | 2 | 3 | 4 | 5 | 6 | 7 |
|---|---|---|---|---|---|---|
| **1 - Calm & Controlled** | | | | | | |
| Agitated, flustered or distracted | | | Just coping ok but feeling challenged | | | Calm, controlled, focused or composed, “just right” |
| **2 - Executing Well** | | | | | | |
| Consumed by “it doesn’t feel right”, “What ifs…?” made me feel indecisive | | | Had to work hard to consciously stay in the present | | | Task focused & able to execute, stay in the moment & remain decisive |
| **3 - Good Anticipation** | | | | | | |
| Behind the play / “half a step off”, often felt sloppy and inaccurate | | | Working hard to keep things simple and to "stay in the game" | | | Anticipating well and ahead of the game, felt precise, accurate, positive |
| **4 - Process Focussed** | | | | | | |
| Stuck in my head / worried about consequences, slow to adapt | | | Being cautious, feeling the pressure, challenged to stay process focussed | | | Task / process focussed / believing in my skills / plan, able to stay in the moment |
| **5 – Prepared** | | | | | | |
| Poorly prepared, unstructured in approach | | | Could only use basic routines with effort | | | Prepared, practised, systematic |
| **6 - Strong Control Systems** | | | | | | |
| Unable to get into my rhythm, use my cues & feel/touch | | | Working hard to find my rhythm and to use my cues & feel/touch | | | Easily able to get into my rhythm and to use my cues & feel/touch to execute well |
| **7 - Balanced & Centred** | | | | | | |
| Unable to get balanced & feel centred/grounded and ready | | | Had to work hard to find balance & feel centred/grounded and ready | | | Easily able to get balanced & feel centred/grounded and ready |
| **8 - Task Focused** | | | | | | |
| Poor task focus, questioning my processes | | | Had to work hard to stay the course & focus on executing my processes | | | Prepared to stay focused on task as long as it takes |
| **9 - Enjoying Progress** | | | | | | |
| Hated my training and felt like I was not progressing as I should | | | Tolerated my training and felt like I was starting to turn the corner | | | Loved my training and consistently felt like I was making progress |

| | | | | | | |
|---|---|---|---|---|---|---|
| **10 – Committed** | | | | | | |
| Gave up easily – felt effort not getting rewarded | | | Consciously working hard at my commitment – beginning to see results | | | Totally committed to my goals & plans, results consistent with my efforts |
| **11 - Self-Driven** | | | | | | |
| I was doing it for others | | | Confused why I was there and who I was doing it for | | | I was doing it just for me |
| **12 – Executing Freely** | | | | | | |
| Important not to fail, worried about possible repercussions of a poor performance | | | Tentative, hesitant, cautious, careful | | | Felt really strong & focussed, excited to execute my processes and perform well |
| **13 – Strategic** | | | | | | |
| Distracted by potential outcomes confused, crooked thinking | | | Challenged to consistently think straight, and focus on executing strategies | | | Enjoyed the strategic challenge of executing my skills under pressure, clear concise thinking |
| **14 – Patient** | | | | | | |
| Unable to slow my thinking, very impatient | | | Developing ability to pace thinking, impatience affecting my ability to control my rhythm & timing | | | Able to think slowly, very patient |
| **15 – Perspective** | | | | | | |
| I was getting stuck on how embarrassing /devastating a poor result could be | | | Some results were harder to deal with from a personal perspective | | | I kept events & outcomes in perspective - "This is what I do, not who I am" |
| **16 - In Control** | | | | | | |
| Often felt I could have prepared better, felt I needed stronger mental performance routines | | | Still developing my belief in my mental and physical preparation / routines | | | Felt my preparation was very good and my performance routines were strong & reliable |
| **17 – Optimistic** | | | | | | |
| Often pessimistic about my ability to thrive under pressure | | | Hoped to be able to cope & perform under pressure | | | Optimistic about my ability to thrive under pressure |
| **18 – Adaptable** | | | | | | |
| Often failed to recognise changing situations | | | Late to adjust to changing situations | | | Able to implement planned responses to changes in performance demands |
| **19 - Thought Control** | | | | | | |
| Regularly having conversations with passing thoughts | | | Still getting distracted by thoughts, having less conversations with them | | | Able to watch thoughts flow by and let go of need for conversations |
| **20 - Self-Belief** | | | | | | |
| Had difficulty bouncing back after making mistakes or errors | | | Could recover ok, but still had difficulty letting go of mistakes | | | Strong belief in my ability to adjust and recover from mistakes, move on |

## Post-Training Instructions

## Rating Scales – introduction

Cognitive Fitness is about how well prepared and "fit for purpose" your thinking systems are for your current challenges.

This set of rating scales are a way for you to identify / score how you have been feeling and how effectively you have been functioning over your last 2 weeks (when training, competing, or at work / when studying).

Use the 7-point rating scales for each of the following aspects of cognitive fitness - there are options for in-between ratings. Choose the <u>number</u> that best represents how you have felt.

# Coach / Assessor Rating Scales for the Pre- and Post-Training Evaluation

**Coach,**
**Give your athlete a score, based on your best knowledge of them, against these tags:**

| Participant ID (Code): | Age: | Gender: | Date: | Coach/Rater: |
|---|---|---|---|---|

| 1 | 2 | 3 | 4 |
|---|---|---|---|
| Focused | Can focus | Distracted | Easily distracted |
| Very sloppy | Sloppy | Accurate | Precise |
| Flowing | Smooth | Stumbling | Balking |
| Decisive | Not afraid of choice | Hesitant | Very hesitant |
| Rigid | Gets stuck | Flexible | Fully adaptive |
| Committed | Persistent | Half-hearted | Gives up easily |
| Balanced | Somewhat balanced | Somewhat imbalanced | Out of balance |
| Very Impatient | Impatient | Patient | Very patient |
| Purposeful | Knows what he/she wants | Unsure about goals | Confused |
| Loves training | Likes training | Tolerates training | Hates training |
| Cruising | Doing well | Doing ok | Struggling |
| Bounces back (from errors, setbacks) | Can recover | Needs help to recover | Unable to recover |
| Not ready for “inner game” training | May be ready | Ready | Natural, fully ready for Cognitive Gym |
| **Last rating above to be replaced with these 2 at Post-Training** | | | |
| Cognitive Gym: embraced it | Cog Gym: didn’t mind it | Cog Gym: did what she/he was told | Cog Gym: did not engage |
| Cognitive Gym: benefited greatly | Cog Gym: some benefit | Cog Gym: little impact | Cog Gym: no use |
