# Supplementary material for: An app-enhanced cognitive fitness training program for athletes: The rationale and validation protocol
Source: Front Psychol. 2022 Aug 30;13:957551. doi: 10.3389/fpsyg.2022.957551 (PMC9469727; doi:10.3389/fpsyg.2022.957551)
Supplement: Supplementary file 2 [file Data_Sheet_2.PDF]

Supplement S2

Typical App screens supporting the Cognitive Gym training program

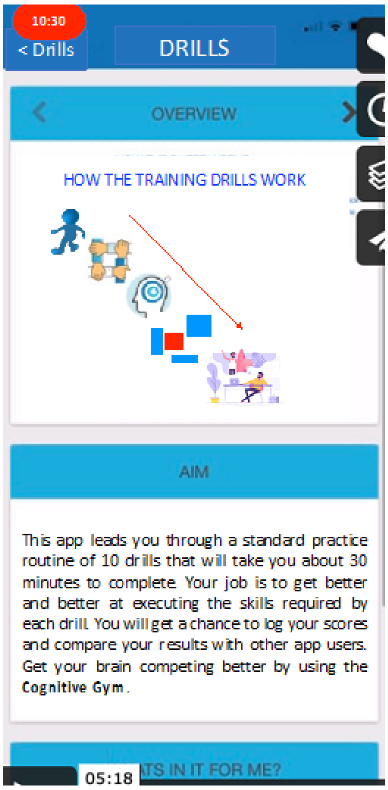

A

Users are introduced to the App by a "home screen" that sets out how the training program will operate and how the app works as a stand-alone training technology

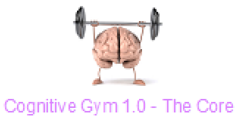

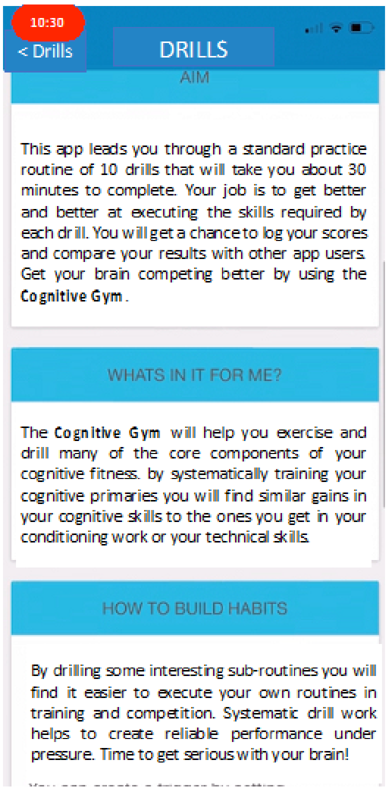

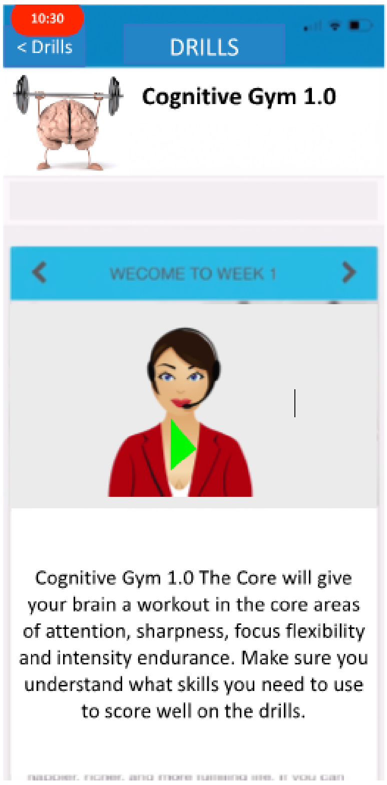

B

Users have access to "how to" guides and tips

They are led through the daily "standard practice routine" by the app, which also records daily completions

i buttons provide reminders or more information for users for each of the drills

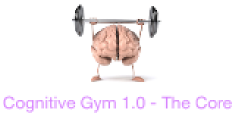

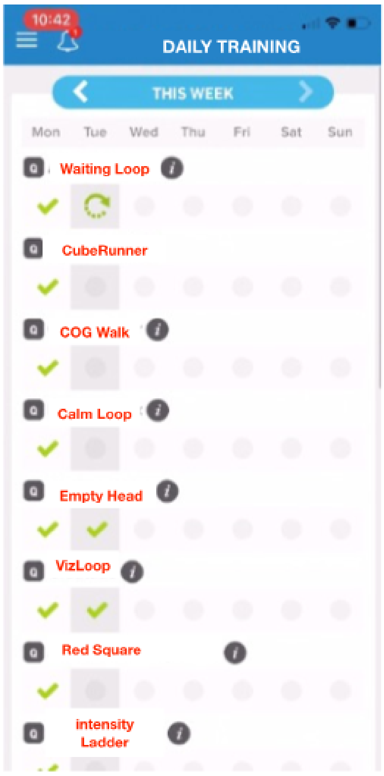

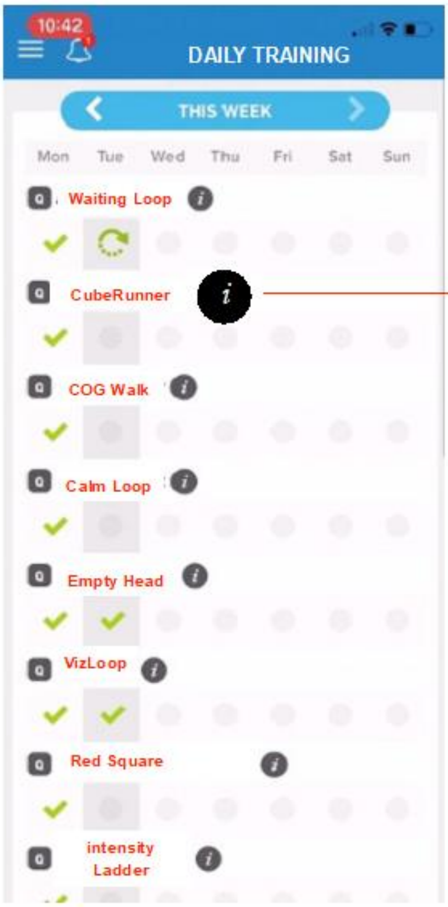

C

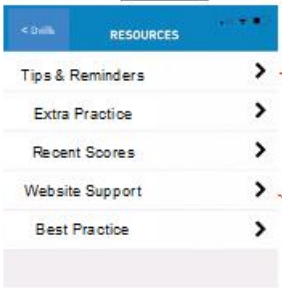

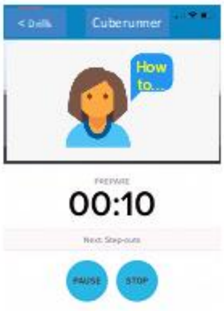

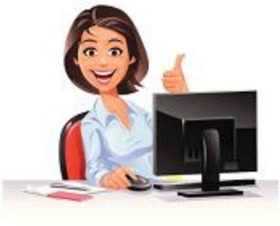

The app assists the user to quickly find additional support & tips and it also can send the user out to the app's support website

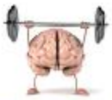

Cognitive Gym 1.0 - The Core

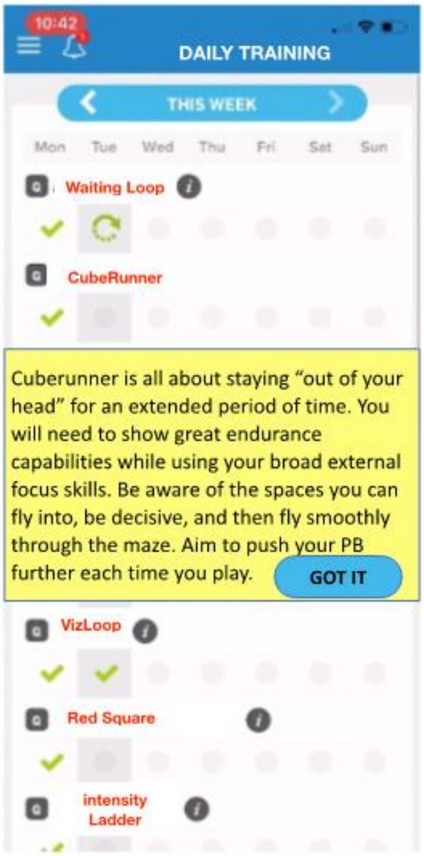

D

i Gives access to further information about the drill

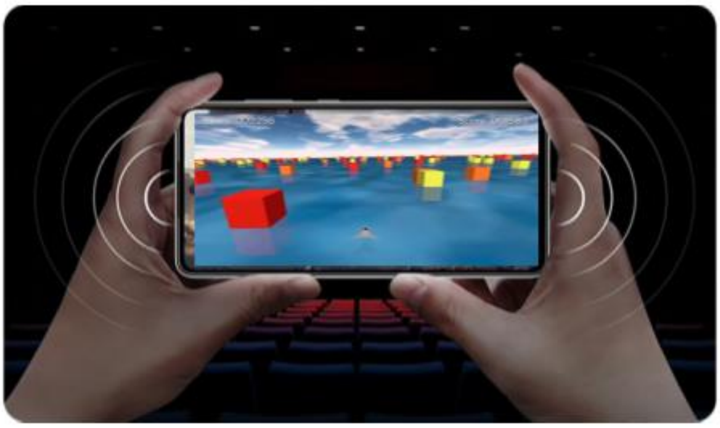

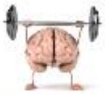

Cognitive Gym 1.0 - The Core

Some drills require the user to set a countdown timer, go to a gamified challenge, then return to the standard practice routine via a notification sent by the app
